# Shaping Food Consumption Among Generation Z in Mexico City: The Role of Digital Stimuli and Brand Engagement in Restaurant Decision-Making

**DOI:** 10.3390/foods15132352

**Published:** 2026-07-02

**Authors:** Iris Leandra Alfonso-Sanjul, Elizabeth Acosta-Gonzaga

**Affiliations:** Sección de Estudios de Posgrado e Investigación, Unidad Profesional Interdisciplinaria de Ingeniería y Ciencias Sociales y Administrativas, Instituto Politécnico Nacional, Av. Té 950, Granjas México, Iztacalco, Mexico City 08400, Mexico; ialfonsos2400@alumno.ipn.mx

**Keywords:** Generation Z, SOR model, food consumption, social media marketing, eWOM, social media influencer, digital marketing, restaurants, purchase intention

## Abstract

Generation Z consumers are reshaping food consumption patterns in urban digital environments, particularly in restaurant contexts characterized by high choice complexity and uncertainty. In Mexico, the evolution of the restaurant industry has intensified the need to understand how digital cues shape consumer food choices. Addressing this gap, this study examines how Social Media Marketing (SMM), Social Media electronic Word of Mouth (Social Media eWOM), and Social Media Influencers (SMIs) shape food consumption intention among Generation Z in Mexico City. Grounded in the Stimulus–Organism–Response (SOR) model and integrating the attitudinal foundations of the Theory of Reasoned Action (TRA) and the Theory of Planned Behavior (TPB), this study analyzes how these digital factors impact food consumption intention (operationalized as restaurant purchase intention) through the mediating psychological mechanism of Consumer Brand Engagement (CBE). A quantitative, non-experimental design was employed using a sample of 406 respondents, and data were analyzed through Structural Equation Modeling (SEM). The results indicate that the model explains 73.6% of the variance in food consumption intention. SMM emerged as the strongest direct predictor, followed by Social Media eWOM and SMIs. Crucially, CBE mediates only the relationship between influencers and consumption intention. Conversely, both SMM and Social Media eWOM exert direct effects that bypass affective engagement. These findings highlight the role of digital ecosystems as cognitive proxies in restaurant selection, providing actionable insights for restaurant SMEs to optimize digital strategies and enhance economic resilience. They also suggest potential implications for healthier and more sustainable urban food environments.

## 1. Introduction

The landscape of micro, small, and medium-sized enterprises (MSMEs) in Mexico between 2019 and 2025 has undergone an unprecedented structural reconfiguration. Following the global health crisis, the MSME sector experienced a period of significant turbulence. According to national statistics, by 2023, while 1.7 million establishments were created, approximately 1.4 million disappeared [1]. Historically, this high mortality rate has been associated with systemic operational deficiencies and persistent misalignment with market demands. Evidence of this challenge is reflected in the 54.0% of existing MSMEs reporting difficulties in customer acquisition, alongside a widespread reluctance to adopt digital channels despite evidence that e-commerce implementation is associated with an 18.3% increase in average annual revenue [2].

Within the food service industry, this gap has been exacerbated by uneven digital adoption among firms. Although innovation in marketing strategies has been recognized as a critical determinant of organizational performance [3], many food-related MSMEs lack structured approaches to managing their digital presence [4]. This limitation becomes critical in an industry where digital content functions as an extension of the food experience itself, shaping expectations prior to purchase. In restaurant settings, where food products function as experience goods whose sensory attributes cannot be evaluated prior to consumption, consumers heavily rely on external digital content and social media cues to mitigate selection risk [5]. Under these circumstances, digital content transcends its traditional promotional role and becomes a strategic mechanism for influencing food consumption behavior [6].

The significance of this phenomenon is particularly evident in Mexico City, where the restaurant industry constitutes one of the most important contributors to national economic activity [7]. As one of the largest and most dynamic urban food markets in Latin America, the city is characterized by a dense and highly diversified gastronomic landscape, intensifying competitive pressures and increasing the complexity of consumer decision-making. Despite these conditions, many establishments continue to exhibit low levels of technological innovation [8], limiting their ability to effectively engage with digitally oriented consumers. In such contexts, strategic market segmentation becomes essential for developing differentiated value propositions that respond to evolving consumption patterns [9].

Among these segments, Generation Z has emerged as a particularly influential driver of change in digital food consumption. As digital natives, their food-related decisions are closely linked to stimuli encountered on social media platforms, where visual, social, and informational cues shape pre-consumption evaluations. In Mexico City, this cohort accounts for approximately 22% of the population [10] and represents the primary user group of food-related digital applications, exerting a broader influence on consumption trends across other demographic segments [11,12]. Recent studies indicate that these consumers actively utilize firm-generated Social Media Marketing (SMM) content to evaluate restaurant offerings [13], rely heavily on peer-generated online reviews within social networks to guide their purchase intentions [14], and are strongly driven by influencers in their buying decisions within the food and beverage sector [15].

Despite the growing relevance of digital stimuli in shaping food-related decision-making, existing research has predominantly examined these phenomena from a general marketing perspective, with limited integration into specialized food consumption frameworks. More specifically, there remains insufficient understanding of how distinct forms of digital stimuli operate through different psychological mechanisms to influence restaurant-related food choices in contexts characterized by uncertainty and strong sensory expectations. Addressing this gap and building upon the key determinants identified in prior literature [16], the present study adopts the Stimulus–Organism–Response (SOR) model while incorporating the conceptual foundations of the Theory of Reasoned Action (TRA) and the Theory of Planned Behavior (TPB). This integrated theoretical approach enables the examination of how external digital marketing stimuli influence consumers’ internal psychological states and, ultimately, their food consumption intentions.

Accordingly, this study pursues the following objectives:

• To examine the direct effects of Social Media Marketing (SMM), Social Media electronic Word of Mouth (Social Media eWOM), and Social Media Influencers (SMIs) on the food consumption intentions of Generation Z consumers within the restaurant sector of Mexico City.
• To evaluate the mediating role of Consumer Brand Engagement (CBE) as an organismic psychological mechanism underlying these relationships.

Ultimately, this research contributes to the literature on digital consumer behavior and food consumption by providing actionable, evidence-based insights for foodservice MSMEs seeking to optimize their digital touchpoints, influence consumer decision-making processes, and strengthen their economic resilience within increasingly competitive urban food markets.

## 2. Literature Review

### 2.1. Generation Z Consumers in the Digital Food Ecosystem

Generation Z includes people born between 1995 and 2009 [17]. Particularly, those living in large cities demonstrate a high level of technological proficiency, which is an inseparable part of their lives [18]. Social media use is very frequent and is considered a typical activity among Generation Z youth. Entertainment, communication, and staying up-to-date are identified as the reasons behind the use of social media platforms [19]. They benefit from a higher volume of information when evaluating product performance and quality [20]. However, they exhibit negative attitudes toward non-organic advertising; they view the so-called ads that appear on these platforms as a hindrance to viewing the information that interests them. In other words, their assessment of the content they consume on social media is contingent on its usefulness to their interests [19].

In the context of food consumption, Generation Z demonstrates a strong reliance on digital environments to evaluate restaurant options prior to consumption. Their decision-making process is highly influenced by visual exposure and peer-generated content, which function as mechanisms to reduce the sensory uncertainty associated with food choice [20]. Gen Z consumers actively seek digital validation through user-generated reviews, high-fidelity images, and influencer content when selecting foodservice establishments [15].

### 2.2. Theoretical Framework

#### 2.2.1. Stimulus–Organism–Response (SOR) Model

Originating in environmental psychology, the SOR model posits that the environment contains stimuli (S) that influence individuals’ internal or organismic states (O), which trigger approach or avoidance responses (R) [21]. This theory has been widely used in consumer behavior research, where digital marketing factors act as strategic environmental stimuli designed to drive purchase intention and customer retention [22,23]. These external signals activate mediating organismic states, such as engagement, which process the received information [13,24,25]. Finally, this internal processing triggers approach-oriented behavioral responses, manifested in the food service industry as purchase intention or visit intention [26,27].

To understand how digital communication influences consumer decision-making, this research is grounded in the Stimulus–Organism–Response (SOR) model. Unlike other static models, the SOR model captures the dynamic nature of digital gastronomic environments, where visual and social stimuli alter the individual’s psychological state prior to the physical transaction [22].

#### 2.2.2. Theory of Reasoned Action (TRA) and Theory of Planned Behavior (TPB)

These theories assume that the best predictor of behavior is intention, determined by attitudes and normative perceptions [28]. The TPB adds perceived control as an additional factor. Previous research has used these approaches to analyze the impact of content and influencer marketing in the food industry [29,30].

Within this framework, the present study partially adopts the attitude construct for the unidimensional measurement of digital marketing factors, recognizing that attitude toward a specific strategy is a strong predictor of purchase intention (Table 1).

### 2.3. Organismic and Behavioral Outcomes

#### 2.3.1. Purchase Intention

Purchase intention is a situational concept that drives consumers to purchase a specific product or service, and generational differences can influence this behavior [31]. The adoption of technological platforms in the hospitality industry is crucial for attracting consumers and influencing their purchasing behavior. Although new factors may emerge in the future, the Internet will remain a fundamental pillar of consumers’ daily lives [32]. Therefore, it is essential that restaurants adapt to technological trends and focus on offering a quality experience to meet consumers’ needs and expectations.

In the context of this study, purchase intention is conceptualized as food consumption intention, referring specifically to the likelihood of consuming food in a restaurant, including the option of ordering online or visiting the establishment.

#### 2.3.2. Engagement

States of consumer engagement occur within a dynamic and iterative process of service relationships that co-create value [33]. In digital marketing, this concept relates to the participation or interaction that a specific strategy is capable of generating. The possible dimensions of engagement in this context can be defined as: following a page, liking content, commenting, and sharing information [34,35].

For its part, Consumer Brand Engagement (CBE) refers to:

“*the cognitive, emotional, and behavioral activity of a consumer, related to the brand and involving a positive evaluation, that occurs during or in relation to specific interactions between the consumer and the brand*”.

[36] (p. 151)

It is a specific form of engagement that occurs between a consumer and a brand, and is therefore appropriate for studies on consumer behavior. Several authors have adopted this form of engagement [37,38]. In this research, when we speak of engagement, we refer to this definition.

In foodservice environments where food functions as an experience good, CBE is critical to processing external cues and forming consumption outcomes. Digital stimuli activate CBE by triggering cognitive interest, positive affect, and brand interaction. Consequently, high levels of psychological engagement effectively mitigate the perceived risks of intangible food attributes, translating virtual interactions into definitive purchase and food consumption intentions [5,34].

### 2.4. Factors Influencing Organismic and Behavioral Outcomes

#### 2.4.1. Social Media eWOM

Electronic Word of Mouth (eWOM) has established itself as the preferred source of information for users, transforming consumer research and decision-making processes [32]. Extremely satisfied or dissatisfied consumers are the most likely to share their experiences. This trend creates an asymmetry in the response, as negative experiences tend to have a greater impact on eWOM than positive ones [39].

eWOM involves two key moments: first, when a user expresses an opinion about a product or service; and second, the subsequent adoption of that information by other consumers. This research focuses on the second moment, that is, on the impact that reviews posted by others have on users’ decisions.

In the restaurant industry, eWOM is a critical factor disseminated through social media and review platforms such as TripAdvisor or Google Maps [14,40,41]. Positive reviews increase the likelihood of consumption and even the willingness to pay higher prices [42].

eWOM on social media platforms enables interaction through comments, generating content that influences brand perception [42]. This is specifically referred to as Social Media eWOM, and its effectiveness depends on the usefulness and relevance of the information to the consumer [43]. Studies in similar contexts indicate that most consumers base their decisions on the eWOM available on social media [14]. In this context, eWOM transcends simple recommendations; it acts as a mechanism for reducing sensory and health risks. Since food is an experiential good, Generation Z uses reviews to validate the quality, hygiene, and visual appearance of the dish, making eWOM a functional validator of the culinary product’s integrity [20,43].

Although the literature sometimes proposes dimensions such as credibility or utility [31], this study measures attitude toward eWOM as a unidimensional construct based on parsimony criteria. A favorable attitude, derived from persuasive and logical messages, is a critical predictor of purchase intention [43,44,45].

##### Relationship Between Social Media eWOM, Engagement, and Purchase Intention

The exchange of opinions online fosters an emotional bond and closeness between consumers and brands [46], strengthening the quality of the relationship [47]. Engagement acts as a key mediator: trust in eWOM increases interaction with the brand, reinforcing purchase intention. This mediating effect has been specifically reported among Gen Z youth and on social media platforms such as TikTok [48,49]. Given that this demographic highly values reviews in the food and beverage sector and based on studies conducted in the Mexican context [50,51,52], the following hypotheses are proposed:

**H1:**  *Social Media eWOM has a positive and significant impact on Generation Z’s purchase intention at restaurants in Mexico City.*

**H1a:**  *Consumer Brand Engagement positively mediates the relationship between Social Media eWOM and Generation Z’s purchase intention at restaurants in Mexico City.*

#### 2.4.2. Social Media Influencers (SMIs)

Social Media Influencers have proven to be key players in shaping purchase intent, especially among younger consumers, thanks to their ability to generate credibility and establish close bonds with their audience [53,54]. Attributes such as attractiveness and reliability determine the effectiveness of their messages [53], varying according to the influencer’s profile: spontaneity among younger influencers and perceived responsibility among older ones [55].

For Generation Z, food bloggers operate as relatable contextual evaluators [56]. The organic response to these stimuli is based on the transfer of trust: the follower delegates the verification of the sensory experience to the influencer [15,53]. However, the vulnerability of this audience underscores the need for ethical principles in such collaborations [39].

This study focuses on attitudes toward SMIs as a unidimensional construct, understood as the follower’s perception and feelings toward the content creator [57]. A positive attitude is directly related to the intention to follow advice and make purchases [57,58].

##### Relationship Between SMIs, Engagement, and Purchase Intention

Engagement acts as a critical mediator; a relationship perceived as warm and similar increases active participation and parasocial interaction [59]. In the food industry, specialized influencers generate positive eWOM and attract new customers through posts about the culinary experience [60]. Various studies confirm that their influence is decisive in purchase intention within the culinary sphere, including in the Mexican context [50,61]. Based on the above, the following hypotheses are proposed:

**H2:**  *Social Media Influencers have a positive and significant impact on Generation Z’s purchase intention at restaurants in Mexico City.*

**H2a:**  *Consumer Brand Engagement positively mediates the relationship between Social Media Influencers and Generation Z’s purchase intention at restaurants in Mexico City.*

#### 2.4.3. Social Media Marketing (SMM)

Digital marketing capabilities, which integrate social media use and e-readiness, are crucial to digital marketing success [62]. Social media is particularly effective at generating interest in new products, services, or brands and driving purchase intention [32,63,64].

In the restaurant industry, Social Media Marketing plays a key role in shaping pre-consumption perceptions of food by providing visual and informational cues. Exposure to food-related images and videos stimulates sensory expectations, influencing consumer attention and appetite prior to purchase [65]. For Generation Z, firm-generated content drives food consumption decisions more effectively than traditional communication channels [13]. The Social Media Marketing variable has been extensively studied in the literature. From a multidimensional perspective, some authors have investigated how its components (interaction, entertainment, personalization, and trends) behave [42,66,67]. However, in accordance with the research objective and parsimony criteria, the variable is analyzed from the unidimensional perspective of attitude toward Social Media Marketing [68,69].

##### Relationship Between SMM, Engagement, and Purchase Intention

Social Media Marketing positively predicts purchase intention [32,64,70], and engagement acts as the mediating variable that strengthens this tendency [35,71]. This phenomenon is particularly evident among Generation Z and in the restaurant industry, where users rely on social media to decide which restaurants to visit [72,73]. In Mexico, the role of these platforms in young people’s purchase intention has been demonstrated [74,75]. Based on the above, the following hypotheses are proposed:

**H3:**  *Social Media Marketing has a positive and significant impact on Generation Z’s purchase intention at restaurants in Mexico City.*

**H3a:**  *Consumer Brand Engagement positively mediates the relationship between Social Media Marketing and Generation Z’s purchase intention at restaurants in Mexico City.*

### 2.5. Proposed Conceptual Model

**Figure 1:**
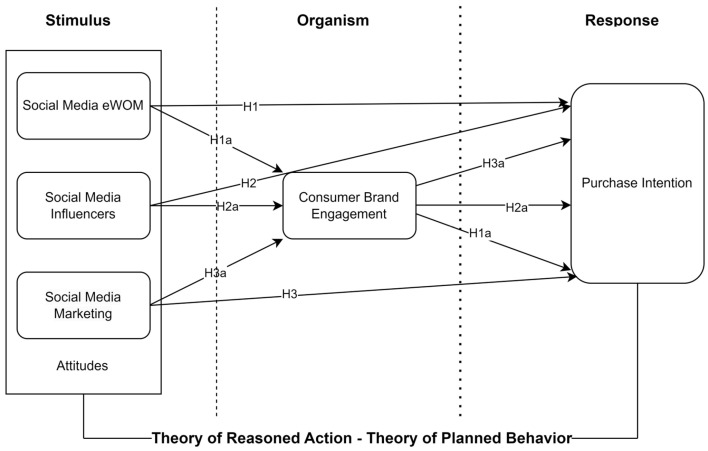
Proposed conceptual model based on the Stimulus–Organism–Response (SOR) model, integrated with the Theory of Reasoned Action (TRA) and the Theory of Planned Behavior (TPB). Abbreviations: eWOM = electronic Word of Mouth. Direct Paths (Stimulus → Response): H1: Social Media eWOM has a positive and significant impact on Generation Z’s purchase intention at restaurants in Mexico City. H2: Social Media Influencers have a positive and significant impact on Generation Z’s purchase intention at restaurants in Mexico City. H3: Social Media Marketing has a positive and significant impact on Generation Z’s purchase intention at restaurants in Mexico City. Indirect Mediating Pathways (Stimulus → Organism → Response): H1a: Consumer Brand Engagement positively mediates the relationship between Social Media eWOM and Generation Z’s purchase intention at restaurants in Mexico City. H2a: Consumer Brand Engagement positively mediates the relationship between Social Media Influencers and Generation Z’s purchase intention at restaurants in Mexico City. H3a: Consumer Brand Engagement positively mediates the relationship between Social Media Marketing and Generation Z’s purchase intention at restaurants in Mexico City.

The conceptual framework of this study integrates the SOR model with the attitudinal foundations of the TRA/TPB (Figure 1). As detailed in the proposed model, three digital marketing factors (SMM, eWOM, and SMIs) act as stimuli (S), operationalized through consumer attitude. These stimuli influence the organismic state (O), represented by engagement, which ultimately triggers the response (R): Generation Z’s purchase intention in the restaurant sector of Mexico City.

This design allows for the exploration of both the direct effects of digital strategies (H1, H2, H3) and the mediating mechanism of engagement (H1a, H2a, H3a), offering a robust approach to understanding digital consumer behavior in Mexico City.

## 3. Materials and Methods

### 3.1. Design

The methodological design of this research is quantitative, non-experimental, cross-sectional, and correlational–causal with mediation analysis, in accordance with the stated objectives and the nature of the phenomenon under study [76]. A pilot test was conducted with 152 Generation Z participants to assess the initial reliability and clarity of the measurement instrument. An exploratory factor analysis (EFA) was performed to identify potential issues in item performance. Based on factor loadings and cross-loading criteria, five items were removed to improve the robustness and parsimony of the scale.

The refined instrument was subsequently validated using confirmatory factor analysis (CFA) in the main study. A structural equation model (SEM) was used to analyze causal relationships.

### 3.2. Measurement Instrument

The exogenous constructs in this research can be multidimensional. However, this study operationalizes SMM, Social Media eWOM, and SMIs as global, unidimensional variables. This uniform approach directly aligns with the objective of this research, which focuses on evaluating the external relationships between the main constructs and their impact on the structural model, rather than dissecting their internal sub-dimensions. It also ensures identical conditions to compare their standardized coefficients within the SEM framework, which aligns with the TRA/TPB attitudinal approach [28]. Furthermore, this parsimonious design kept the self-administered questionnaire concise for Generation Z, preventing cognitive fatigue and ensuring data reliability.

The instrument was developed using scales previously validated in scientific literature (Table 2). Specifically, items for Social Media eWOM and Purchase Intention were derived from Esparza-Huamanchumo et al. [14] and Park et al. [44]. The Social Media Influencers scale was based on Chetioui et al. [77], while the Social Media Marketing indicators were adapted from Zhang et al. [69] and Durukan and Bozaci [78]. Finally, Consumer Brand Engagement was operationalized using the scale developed by Hollebeek et al. [36]. The items were translated into Spanish and adapted to the context of this study. A 1- to 5-point Likert scale was used for measurement, with 1 equating to “definitely no” and 5 to “definitely yes.” Additionally, the questionnaire included an initial section focused on collecting sociodemographic information from the participants, using a “select the correct answer” format. This section asks about gender, current activities (school, work, etc.), frequency of dining out, and preference for online or in-person dining.

### 3.3. Study Participants

#### 3.3.1. Sample Calculation

The study targets Generation Z youth, who number 2,117,283 according to the most recent census [10]. However, the study population consists of those Generation Z youth who dine out at restaurants. Since this specific data is unknown, the sample size was calculated based on an indeterminate population framework. The parameters were then defined using a 95% confidence level and a 5% margin of error, in accordance with established statistical constraints [79]:

n = (Z^2^ · p · q)/d^2^

where

• n = sample size;
• Z = confidence level (95%);
• p = expected proportion (0.5);
• q = 1 − p (0.5);
• d = margin of error (0.05).

Substituting the values, we obtained: n = ((1.96)^2^ · 0.5 · 0.5)/(0.05)^2^ = 384.16.

Therefore, an adequate sample size of 385 participants who meet the defined profile is established.

#### 3.3.2. Data Collection Process

Data collection took place in November 2025 using Google Forms, targeting university students. To distribute the questionnaire among students who met the study’s criteria, support was requested from professors at the Unidad Profesional Interdisciplinaria de Ingeniería y Ciencias Sociales y Administrativas (UPIICSA) of the Instituto Politécnico Nacional (IPN). Additionally, in-person surveys were conducted on campus by providing a QR code, enabling participants to digitally access and complete the questionnaire. In this way, a total of 456 responses were obtained.

#### 3.3.3. Inclusion Criteria

To ensure strict adherence to the target population, two mandatory inclusionary criteria were operationalized via screening questions positioned at the baseline of the digital instrument:

• Belonging to Generation Z and being of legal age at the time of data collection, establishing a range between 18 and 30 years old.
• Having dined at a restaurant in Mexico City within the preceding 6 months.

Of the 456 initial responses gathered during the fieldwork, 50 questionnaires did not satisfy the inclusion criteria and were subsequently excluded from the dataset.

**Figure 2:**
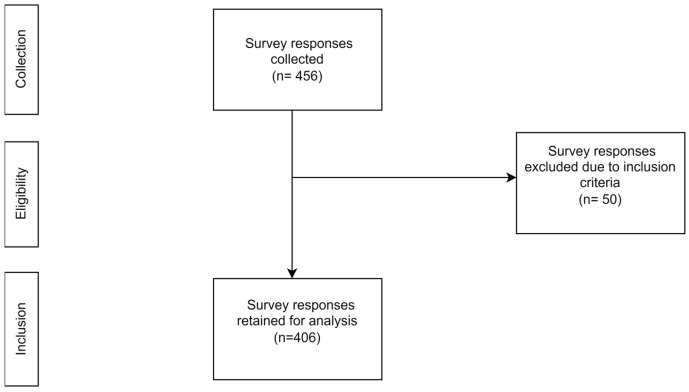
Flowchart of the sampling process across three stages: Collection, Eligibility, and Inclusion. It maps the progression from initial responses (n = 456) through the exclusion of cases that failed the screening criteria (n = 50), resulting in the final sample (n = 406) retained for quantitative analysis. Cases were excluded if they did not meet the exact inclusion criteria: (1) belonging to Generation Z and being of legal age at the time of data collection (18 to 30 years old), and (2) having dined at a restaurant in Mexico City within the preceding 6 months. Source: Author’s own work.

Consequently, a final valid sample of 406 cases was retained for the structural equation modeling (SEM) analysis, strictly exceeding the minimum statistical threshold of 385 participants required by the sampling design. The complete screening process is detailed in Figure 2.

## 4. Analysis and Results

### 4.1. Descriptive Analysis

The gender distribution is balanced, consisting of 50.5% men and 49.5% women. Regarding their occupation, 66.5% are exclusively dedicated to their studies, while an additional 24.8% combine their studies with employment or entrepreneurship (Table 3).

Regarding their dining habits, the most common frequency of consumption is monthly (28.6%), followed by weekly (22.4%). Despite being digital natives, they maintain a strong preference for the in-person experience; 70.7% of respondents prefer to visit the establishment directly, while the remaining 29.3% opt for online ordering.

### 4.2. Confirmatory Factor Analysis

The measurement model, consisting of five latent factors (eWOM, SMI, SMM, CBE, and PI) and 25 observed variables, was evaluated using the Maximum Likelihood (ML) method. Following an iterative process, items SMM3 and SMM4 were eliminated because their factor loadings were lower than 0.70.

Furthermore, following the recommendations of Brown [80], a covariance between error terms was incorporated between items CBE1 and CBE2. These items belong to the same subdimension of the CBE concept [36]. This practice is methodologically justified in the literature [80] provided that the variables are not under-identified (have fewer than 3 items) and there is a theoretical justification for the similarity in wording, as explained above.

### 4.3. Model Fit

The goodness-of-fit indices (Table 4) demonstrate that the proposed measurement model fits the empirical data adequately, exceeding the thresholds suggested by Hooper et al. [81].

The value and an RMSEA of 0.070 confirm minimal discrepancy and low approximation error. Meanwhile, the incremental indices (CFI and TLI) close to 0.95 reflect a robust fit that minimizes the risk of overfitting.

### 4.4. Convergent Validity

Convergent validity was confirmed by examining the standardized factor loadings. All remaining items had significant coefficients greater than 0.70, meeting the criteria of Hair et al. [82] to ensure the internal consistency of the constructs (Table 5).

As shown in Table 5, the correlation between Social Media eWOM and Consumer Brand Engagement (CBE) yielded a coefficient of −0.050. Methodologically, a correlation coefficient with an absolute value below 0.10 represents an effect size smaller than a small effect and is generally interpreted as reflecting a negligible association [83,84]. Therefore, the value of −0.050 does not reflect a meaningful negative structural pattern, nor does it imply a theoretical contradiction.

To ensure that each construct in the model represents a unique concept distinct from the others, discriminant validity was assessed using the Fornell & Larcker criterion [85]. This method stipulates that the square root of the Average Variance Extracted (AVE) for each latent variable must be greater than the correlations it shares with any other construct in the system. As shown in Table 6, the values on the diagonal (highlighted in bold) represent the square root of the AVE, while the values off the diagonal represent the correlations between the variables.

### 4.5. Structural Equation Modeling (SEM)

Once the scale was validated, Structural Equation Modeling (SEM) was performed to test the causal hypotheses regarding Generation Z’s purchase intention (PI) in the restaurant sector of Mexico City.

**Figure 3:**
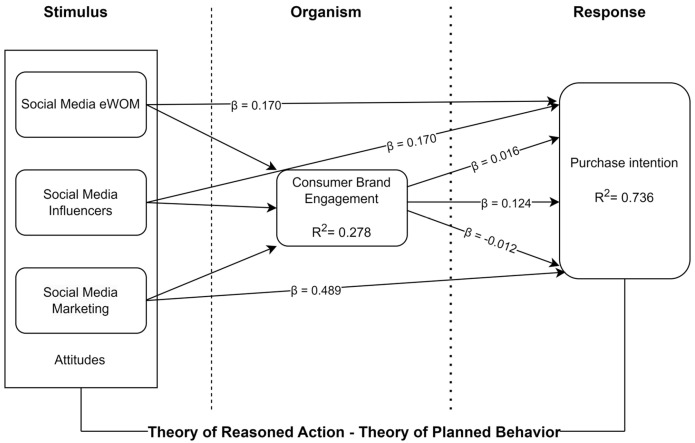
Empirical results of the structural equation modeling (SEM) analysis for the proposed framework. The diagram displays the standardized path coefficients (β) and coefficients of determination (R^2^) for each structural relationship.

The model yields an R^2^ of 0.736. This indicates that Social Media Marketing, Influencers, eWOM, and Engagement collectively explain 73.6% of the variance in Gen Z’s purchase intention in Mexico City. Engagement (CBE) yielded an R^2^ of 0.278 (Figure 3).

To evaluate the mediation hypotheses (H1a, H2a, and H3a), the bootstrapping technique was applied with 2000 subsamples and a 95% confidence interval. The results of the path analysis are summarized in Table 7.

## 5. Discussion

### 5.1. Direct Predictors of Purchase Intention

The model explains 73.6% of the variance (R^2^ = 0.736) in purchase intention, providing strong empirical evidence of how digital stimuli shape food consumption behavior in restaurant contexts. This high predictive power supports the integration of the Stimulus–Organism–Response (SOR) model with the attitudinal foundations of the Theory of Reasoned Action (TRA) and the Theory of Planned Behavior (TPB).

Social Media Marketing (SMM) emerges as the strongest predictor of purchase intention, exhibiting the highest standardized effect in the structural model (β = 0.489; *p* = 0.001). This finding aligns with other research on the centrality of firm-generated digital strategies in shaping consumer behavior in online environments [5,72,86]. Particularly within the food sector, high exposure to digital platforms significantly drives product recognition and consumer interest through memorable visual and promotional cues [19]. Accordingly, SMM not only enhances brand awareness [64], but also functions as a stimulus mechanism for attracting consumers to restaurants [32].

Social Media eWOM exhibits a positive and statistically significant effect on purchase intention (β = 0.170; *p* = 0.005). Young restaurant consumers rely heavily on online reviews to validate consumption decisions [14,42]. This behavior reflects a reliance on peer-generated recommendations and socially endorsed information prior to visiting an establishment [32]. However, its effect is weaker than that of SMM, which may be explained by asymmetries in review processing. As suggested by Kim and Hwang [39], negative information tends to exert a stronger influence than positive information due to negativity bias. Consequently, eWOM primarily functions as a filtering mechanism, helping consumers evaluate price, location, and experiential attributes based on others’ subjective experiences [87]. Its effectiveness is further influenced by the perceived anonymity of the source and skepticism regarding the commercial intentions of reviewers [20,43].

Social Media Influencers (SMI) also exert a positive and significant effect on Generation Z’s purchase intention (β = 0.170; *p* = 0.001). This finding supports the role of influencers as credible social actors within digital ecosystems, where users actively seek guidance from perceived members of online communities [60]. In food-related contexts, this influence is particularly relevant as influencers act as experiential proxies, enabling consumers to vicariously experience the sensory attributes of food, through visual and narrative content. Nevertheless, their influence remains moderate relative to SMM, consistent with Flores et al. [88], who suggest that influencer content is often perceived as entertainment rather than as an authoritative decision-making source. Despite this, the perceived utility of food bloggers’ recommendations substantially influences attitude and visit intention [60], particularly when delivered through visually rich formats such as images and short-form videos [54]. In the Mexican Generation Z context, celebrities and influencers remain effective attention drivers [61]; however, overtly promotional strategies such as discounts or gifted collaborations may generate skepticism regarding product authenticity and quality, thereby limiting persuasive effectiveness [54].

### 5.2. Mediation of Consumer Brand Engagement (CBE)

A key contribution of this study is the examination of the mediating role of Consumer Brand Engagement (CBE). The construct exhibits an R^2^ of 0.278, indicating a moderate level of explained variance in the endogenous mediator.

The indirect effect of Social Media Influencers (SMIs) on purchase intention (β = 0.124) confirms that CBE plays a significant mediating role in this relationship. This finding supports the argument that perceived similarity, relational closeness, and affective congruence with influencers enhance consumer engagement and persuasive effectiveness [55,59]. Young consumers tend to view influencers as peers, establishing parasocial bonds rooted in perceived friendship [55]. This closeness strengthens the brand’s credibility and transforms followers into brand advocates within their social networks [56]. The influencer’s reputation, particularly on visually oriented platforms such as TikTok, further strengthens both cognitive and emotional engagement processes [15], reinforcing their role as relational intermediaries in consumer decision-making [53,54]. From an ethical perspective, these dynamics also highlight the need for transparency and accuracy in influencer communications, given the susceptibility of young consumers to persuasive opinion leadership effects [55].

The structural model does not support the mediating role of Consumer Brand Engagement (CBE) in the relationships between either Social Media Marketing (SMM) or Social Media eWOM and purchase intention.

Conversely, SMM emerged as the strongest direct predictor within the structural system. This empirical outcome challenges previous marketing literature, which asserts that firm-generated digital activities must operate through cognitive and affective engagement processes before influencing behavioral intention [42,70]. This phenomenon is highly contextualized by the nature of the restaurant industry, where food products exhibit characteristics of experience goods that cannot be fully evaluated prior to consumption. For Generation Z consumers in Mexico City, firm-generated content—such as high-resolution food imagery, menu updates, and real-time promotional information—functions as a primary diagnostic [63]. Rather than engaging in relational or interactive processing [36], young consumers interpret official brand content as an immediate indicator of quality, availability, and expected experience.

The same immediate, non-relational processing explains the lack of mediation for Social Media eWOM. This behavior confirms that perceived usefulness and informational quality act as the primary drivers of eWOM adoption [20,33]. Simply learning about others’ real experiences and gaining a clear idea of the food’s taste and attributes is sufficient for prospective consumers to decide whether to visit the restaurant [89], rendering a deeper relational bond unnecessary.

The distinction between direct cognitive pathways and engagement-mediated affective pathways can be explained through the theoretical lens of Parasocial Interaction Theory [90]. Grounded in this perspective, Social Media Influencers represent a humanized social stimulus where the individual remains the absolute center of the interaction [91,92]; because the source is a human actor, consumers form one-sided relationships based on social trust, demanding the relational mediation of CBE to translate parasocial closeness into brand commitment. Conversely, SMM is predominantly perceived as institutional and transactional in nature, while eWOM operates as decentralized peer validation. As highlighted by Labrecque [93], when social media stimuli lack a humanized focal creator as their core or are perceived as strictly commercial and automated, they fail to trigger strong parasocial attachment mechanisms. Consequently, these non-parasocial stimuli drive rapid cognitive choices that bypass affective filters and rely strictly on the objective utility of the data.

### 5.3. Practical Implications and Recommendations for Restaurant MSMEs

To maximize the utility of these findings for Micro, Small, and Medium Enterprises (MSMEs) in the Mexican restaurant sector, which frequently operate under strict financial, technological, and staffing constraints [2,4], generic marketing mandates must be translated into low-cost operational tactics.

Given the dominance of the direct Social Media Marketing (SMM) pathway (β = 0.489, *p* = 0.001), owners and communication professionals can achieve high-fidelity sensory validation using affordable consumer technologies, ensuring that firm-generated activities target the immediate informational needs of the consumer [13]. Specifically, MSME restaurants should prioritize the creation of unedited, short-form video content, such as TikToks or Instagram Reels, focused on close-up captures of food preparation, steam, and portion sizes. Because official brand content serves as a direct benchmark for Gen Z’s consumption choices [29], maintaining an updated digital menu (pinned, easily accessible, with transparent pricing, and real-time availability) acts as a key informational signal that facilitates purchase conversion. Furthermore, integrating direct messaging channels, such as WhatsApp Business, streamlines communication and actively supports immediate purchase intention.

Furthermore, because Generation Z consumers utilize online reviews as a practical filter for decision-making, hospitality firms should actively stimulate user-generated reviews across digital platforms. To achieve this, QR codes can be strategically placed on tables, receipts, or loyalty cards with explicit invitations for diners to rate their experience. Concurrently, service staff can be trained to actively invite visibly satisfied customers to leave a review or comment. Providing timely and empathetic responses to negative feedback is also essential, as visible service recovery signals operational reliability to potential consumers who rely on digital platforms before selecting a restaurant.

Finally, social media influencer (SMI) campaigns should be redefined as long-term relational strategies rather than immediate sales tools, as their strategic impact depends directly on user engagement. To mitigate the financial constraints typical of Mexican MSMEs, it is highly viable to explore collaborations with local gastronomic micro-influencers within Mexico City. Aggressive promotional discounts should be avoided, as they may inadvertently compromise perceived food quality. Instead, these creators should be invited to co-create vertical video content published on either the restaurant’s or the influencer’s profile. Documenting the authentic service experience, kitchen preparation, and overall atmosphere allows followers to assimilate the content as a legitimate peer recommendation.

### 5.4. Potential Implications for Food Security, Sustainable Consumption and Nutritional Outcomes

Beyond their managerial implications, these findings are also relevant from a broader urban food systems perspective. In Mexico City, restaurants are not merely commercial establishments but also important spaces where gastronomic heritage, cultural identity, and contemporary consumption practices intersect. Digital marketing strategies therefore, influence not only restaurant choice but also the visibility and preservation of local culinary traditions, contributing to the dynamic evolution of Mexico City’s gastronomic culture [94]. At the same time, the growing influence of digital stimuli on Generation Z’s food choices highlights the opportunity for restaurants to promote healthier and more sustainable consumption patterns through responsible communication. Given that many popular dishes sold in Mexico City’s food environment are high in energy, fat, and sodium [95], digital platforms can be used to increase the visibility of healthier menu options, transparent nutritional information, and balanced meal alternatives. Such practices are consistent with recent evidence showing that healthier and more sustainable dietary patterns in Mexico can simultaneously reduce environmental impacts and improve nutritional quality, particularly in large urban areas such as Mexico City [96,97]. More broadly, these findings suggest that digital restaurant marketing has implications beyond firm competitiveness, as it may contribute to food security and sustainable urban food systems by encouraging dietary choices that align with long-term public health and environmental objectives [98,99]. Consequently, responsible digital communication should be viewed as a complementary mechanism through which restaurant MSMEs can simultaneously strengthen economic resilience while supporting healthier and more sustainable urban food environments.

## 6. Conclusions, Limitations and Future Research

This study examined how digitally mediated stimuli shape food consumption behavior in restaurant contexts, with particular emphasis on Generation Z consumers in Mexico City. By conceptualizing purchase intention as food consumption intention, the study provides a more contextually grounded understanding of how consumers evaluate and select restaurants based on digital stimuli.

From a theoretical perspective, this study contributes by integrating the Stimulus–Organism–Response (SOR) model with the attitudinal foundations of the Theory of Reasoned Action (TRA) and the Theory of Planned Behavior (TPB) within contemporary digital food environments. A key theoretical contribution lies in the empirical validation that incorporating attitudinal constructs within the SOR architecture offers a parsimonious yet powerful explanation of behavioral intention in digitally mediated consumption contexts. Furthermore, the findings demonstrate that digitally mediated stimuli do not operate homogeneously through the consumer’s internal psychological processes. While electronic Word of Mouth (eWOM) and Social Media Marketing (SMM) were traditionally assumed to require emotional or interactive engagement to manifest in behavior, the structural model reveals that these stimuli bypass the affective mechanisms of Consumer Brand Engagement (CBE). Conversely, Social Media Influencers (SMIs) still heavily rely on CBE as a relational mediator to transform visual exposure into definitive food consumption intentions. From a practical standpoint, these results highlight the need for restaurant MSMEs to strategically manage digital touchpoints that shape consumer perceptions prior to consumption.

Overall, this study contributes to the literature on food consumption behavior by demonstrating how digital environments reshape the way consumers evaluate restaurants prior to consumption. In contexts characterized by uncertainty and limited direct experience, digitally mediated stimuli become critical drivers of decision-making, particularly among younger consumers in complex urban food systems.

Despite the contributions of this study, it is important to acknowledge certain limitations that constrain the scope of the findings. First, the sample was drawn exclusively from a single university (UPIICSA-IPN) via convenience sampling; although the calculated sample size exceeded, this narrow sampling frame limits the generalizability of the results to the broader, socioeconomically diverse Generation Z population of Mexico City. Second, although the study focuses on the MSME ecosystem, it does not differentiate between firm size variations or restaurant typologies, nor does it account for differences in food categories (e.g., healthy, indulgent, or sustainable offerings), which may moderate consumer responses. Third, the reliance on self-reported data introduces potential common method bias and subjectivity effects. Finally, as a fourth limitation, the use of unidimensional constructs, adopted for model parsimony, limits the capture of multidimensional behavioral nuances.

Future research is encouraged to employ multidimensional measurement models, expand sampling frames to include more heterogeneous populations, and examine whether digital stimuli exert differential effects across restaurant types and dietary or ethical consumption contexts. Also, future studies could evaluate the potential implications regarding food security, sustainable consumption, and nutritional outcomes proposed in this study, specifically focusing on how responsible digital communication can promote healthier dietary patterns within this urban food environment. Finally, future research should transition to the post-consumption stage, integrating core hospitality factors such as food and service quality alongside digital marketing metrics to evaluate their impact on customer satisfaction, brand equity, and re-purchase decisions.

## Figures and Tables

**Table 1:** Comparison of theories and decision on application.

Theoretical Model	Approach	Relation to the Study	Decision
SOR	Stimuli, environment, internal states, response.	Analyze how digital marketing generates engagement and purchase intent.	Selected (Main framework).
TRA/TPB	Attitude and norms as predictors of intention.	Provides the theoretical basis for measuring strategies through attitude.	Partially adopted (Operationalization).

**Table 2:** Operationalization of constructs.

Construct	Items	Source
Social media eWOM (Attitude toward eWOM)	Regarding COMMENTS and REVIEWS of restaurants on social media.	Esparza-Huamanchumo et al., 2024; Park et al., 2007 [14,44]
	1.1 I always check them to decide which restaurant to go to.	
	1.2 They give me confidence to visit a restaurant.	
	1.3 They help me make a better decision about which restaurant to go to.	
Social Media Influencers (Attitude toward Social Media Influencers)	Regarding FOOD INFLUENCERS (or foodies) on social media.	Chetioui et al., 2020 [77]
	2.1 They serve as a guide for me.	
	2.2 Their content is interesting.	
	2.3 They keep me informed about restaurants and let me know about promotions and deals.	
	2.4 I trust them to discover and provide reliable information.	
Social Media Marketing (Attitude toward Social Media Marketing)	Regarding the MARKETING that restaurants do on social media.	Zhang et al., 2022; Durukan & Bozaci, 2012 [69,78]
	3.1 Marketing a restaurant’s offerings (such as dishes or experiences) on social media is appealing.	
	3.2 It’s a good idea for restaurants to market themselves on social media	
	3.3 All restaurants should engage in marketing on TikTok.	
	3.4 All restaurants should engage in marketing on Facebook.	
	3.5 All restaurants should engage in marketing on Instagram.	
	3.6 I like the marketing activities carried out on social media.	
	3.7 Restaurants should use social media for marketing.	
	3.8 I believe that social media marketing will be the future of marketing for restaurants.	
Consumer Brand Engagement	When I see content from Restaurant X on social media (reviews, posts, recommendations from influencers).	Hollebeek et al., 2014 [36]
	4.1 It makes me happy.	
	4.2 I feel good when I see content from Restaurant X on social media.	
	4.3 I feel proud to follow or interact with content from Restaurant X.	
	4.4 I spend a lot of time viewing content from Restaurant X compared to other restaurants.	
	4.5 When I use social media, I usually look for or check out content about Restaurant X.	
	4.6 X Restaurant’s social media account is one of the ones I visit most often when I’m browsing social media.	
Purchase Intention	After seeing or reading content about a restaurant on social media (reviews, posts, recommendations from influencers).	Esparza-Huamanchumo et al., 2024 [14]
	5.1 It is very likely that I will dine at that restaurant.	
	5.2 If I want to, I’ll order a dish at that restaurant.	
	5.3 I will definitely try a dish from that restaurant.	
	5.4 I will recommend that restaurant to other people.	

**Table 3:** Sociodemographic profile and consumption behavior of the participants.

Variable	Category	Frequency (n)	Percentage (%)
Gender	Male	203	50.0
	Female	199	49.0
	Prefer not to say	4	1.0
Occupation	Student (only)	270	66.5
	Student and Employee	57	14.0
	Student and Entrepreneur	44	10.8
	Employee (only)	15	3.7
	Others	20	4.9
Consumption Frequency	Monthly	116	28.6
	Weekly	91	22.4
	Every two weeks	74	18.2
	Two or more times per week	64	15.8
	Every two months or more	61	15.0
Consumption Mode	On-site (In-person visit)	287	70.7
	Online (Delivery/Apps)	119	29.3

The “Others” category includes “Entrepreneur,” “Housewife,” and combined roles representing less than 2% of the sample.

**Table 4:** Overall fit indices of the measurement model.

Fit Index	Model Value	Recommended Criterion
Chi-square/Degrees of Freedom	(χ^2^/df), 2.973	<3.0
Comparative Fit Index (CFI)	0.937	≥0.90
Tucker–Lewis Index (TLI)	0.927	≥0.90
Root Mean Square Error of Approximation (RMSEA)	0.070	≤0.08

**Table 5:** Construct validity through composite reliability and the mean of the variance extracted.

Latent Factor	Cronbach’s Alpha (α)	CR	AVE	Observed Variable	Factor Loading (β)	Estimate (B)	Standard Error (S.E.)
Social Media eWOM	0.865	0.866	0.684	EWOM1	0.771	1.018	0.058
				EWOM2	0.844	0.982	0.050
				EWOM3	0.857	1.000	---
Social Media Influencers	0.865	0.864	0.614	SMI1	0.786	1.016	0.062
				SMI2	0.771	0.881	0.055
				SMI3	0.761	0.951	0.060
				SMI4	0.803	1.000	---
Social Media Marketing	0.925	0.927	0.680	SMM1	0.720	0.841	0.052
				SMM2	0.888	1.089	0.050
				SMM5	0.793	0.998	0.055
				SMM6	0.813	0.957	0.050
				SMM7	0.893	1.109	0.051
				SMM8	0.809	1.000	---
Consumer Brand Engagement	0.932	0.933	0.638	CBE1	0.713	0.690	0.040
				CBE2	0.707	0.676	0.039
				CBE3	0.824	0.910	0.041
				CBE4	0.892	1.000	---
				CBE5	0.859	1.016	0.042
				CBE6	0.874	1.035	0.041
Purchase Intention	0.865	0.865	0.615	PI1	0.801	1.000	---
				PI2	0.775	0.948	0.056
				PI3	0.788	1.031	0.060
				PI4	0.819	1.062	0.059

**Table 6:** Discriminant validity analysis.

Construct	Social Media eWOM	SMI	SMM	CBE	PI
Social Media eWOM	**0.827**				
Social Media Influencers	0.453	**0.783**			
Social Media Marketing	0.724	0.569	**0.825**		
Consumer Brand Engagement	−0.050	0.512	0.064	**0.799**	
Purchase Intention	0.170	0.170	0.489	0.243	**0.784**

Note: Values on the diagonal in bold represent the square root of the Average Variance Extracted (AVE), while off-diagonal values represent inter-construct correlations.

**Table 7:** Summary of significant relationships in the structural model.

Hypothesis	Path	Coef β	Result
H1	Social Media eWOM → PI	β = 0.170	H1 = Accepted (*p* = 0.005)
H2	SMI → PI	β = 0.170	H2 = Accepted (*p* = 0.001)
H3	SMM → PI	β = 0.489	H3 = Accepted (*p* = 0.001)
H1a	Social Media eWOM → CBE → PI	β = −0.012	H1a = Rejected (*p* = 0.501)
H2a	SMI → CBE → PI	β = 0.124	H2a = Accepted (*p* = 0.001)
H3a	SMM → CBE → PI	β = 0.016	H3a = Rejected (*p* = 0.455)

## Data Availability

The original contributions presented in this study are included in the article. Further inquiries can be directed to the corresponding author.

## Abbreviations

The following abbreviations are used in this manuscript:

MSMEs	Micro, Small, and Medium-Sized Enterprises
SMM	Social Media Marketing
eWOM	Electronic Word of Mouth
SMI	Social Media Influencers
CBE	Consumer Brand Engagement
SOR	Stimulus–Organism–Response
TPB	Theory of Planned Behavior
TRA	Theory of Reasoned Action
CFA	Confirmatory Factor Analysis
SEM	Structural Equation Modeling

## References

[1] INEGI (2024). Estudio Sobre la Demografía de los Negocios (EDN).

[2] ASEM (2025). Radiografía del Emprendimiento en México 2025.

[3] Cerón T., Morales Y., Santiesteban N. (2021). Estrategias de Mercadotecnia Gastronómica para Restaurantes de Puebla en Tiempos de Pandemia por COVID-19. Estudio de Caso: Generación Z. Eur. Sci. J. O Eur. Sci. J..

[4] González-Compeán R.O., Rangel-Lyne L., Ochoa-Hernández M.L. (2024). Marketing digital en microempresas de servicios alimentarios. Beneficios, intención de uso y la nula moderación de la formalidad. Estud. Soc. Rev. Aliment. Contemp. Y Desarro. Reg..

[5] Ali-Alsaadi A.A., Cabeza-Ramírez L.J., Sántos-Roldán L., Loor-Zambrano H.Y. (2023). Digital Marketing and Fast-Food Intake in the UAE: The Role of Firm-Generated Content among Adult Consumers. Foods.

[6] Chávez D., Cavazos J. (2024). Efectos de la innovación en marketing sobre valor percibido y engagement del consumidor en restaurantes de una operadora en Ciudad de México. Contaduría Y Adm..

[7] INEGI (2021). Censos Económicos 2019: La Industria Restaurantera en México.

[8] Arellano Narváez R., Acosta Gonzaga E. (2020). Use of delivery service apps in newly created gastronomic microenterprises in Mexico City. Adm. Y Organ..

[9] Clarke A.H., Freytag P.V., Mora Cortez R. (2024). Revisiting the strategic role of market segmentation: Five themes for future research. Ind. Mark. Manag..

[10] SEDECO (2020). Principales Resultados del Censo Población y Vivienda 2020.

[11] Kotler P., Kartajaya H., Setiawan I. (2021). Marketing 5.0 Versión México: Tecnología Para la Humanidad.

[12] Lara J., Cervantes J. (2021). Social media como herramienta de comunicación para los integrantes de la Generación Z: Caso COVID-19. Tópicos del Marketing.

[13] Anas A.M., Abdou A.H., Hassan T.H., Alrefae W.M.M., Daradkeh F.M., El-Amin M.A.-M.M., Kegour A.B.A., Alboray H.M.M. (2023). Satisfaction on the driving seat: Exploring the influence of social media marketing activities on followers’ purchase intention in the restaurant industry context. Sustainability.

[14] Esparza-Huamanchumo R.M., Quiroz-Celis A.V., Camacho-Sanz A.A. (2024). Influence of eWOM on the purchase intention of consumers of Nikkei restaurants in Lima, Peru. Int. J. Tour. Cities.

[15] Sang V.M., Duy Khuong B., Yen Nhi L., Gia Han L., Minh Giau T., Thi Tuyet Ngoan N.T. (2024). The impact of food reviewers on purchase intention in the food and beverage industry: The mediating role of user interaction. Cogent Bus. Manag..

[16] Alfonso-Sanjul I.L., Acosta-Gonzaga E. (2026). Digital marketing factors influencing Generation Z’s purchase intention in the restaurant sector: A systematic review. ECORFAN J. Mex..

[17] McCrindle Generation Z—The Future Consumer. McCrindle. https://mccrindle.com.au/article/topic/generation-z/generation-z-the-future-consumer/.

[18] Gautama M., Budiman G.I., Zuraida R. (2023). Consumer Satisfaction and Purchasing Behavior Through Online Food Delivery Services App. Proceedings of 2023 International Conference on Information Management and Technology, ICIMTech 2023, Malang, Indonesia, 24–25 August 2023.

[19] Ares G., Antúnez L., de León C., Alcaire F., Vidal L., Natero V., Otterbring T. (2022). “Even if you don’t pay attention to it, you know it’s there”: A qualitative exploration of adolescents’ experiences with digital food marketing. Appetite.

[20] Ngo T.T.A., Vuong B.L., Le M.D., Nguyen T.T., Tran M.M., Nguyen Q.K. (2024). The impact of eWOM information in social media on the online purchase intention of Generation Z. *Cogent Bus*. Manag..

[21] Mehrabian A., Russell J.A. (1974). An Approach to Environmental Psychology.

[22] Zaib A., Qummar H., Bashir S., Aziz S., Hooi D. (2024). Customer engagement in Saudi food delivery apps through social media marketing: Examining the antecedents and consequences using PLS-SEM and NCA. J. Retail. Consum. Serv..

[23] Ngo T.T.A., An G.K., Dang N.Y., Doan T.T., Nguyen V.M.H. (2026). The psychological impact of social media marketing on consumer willingness to pay for tech gadgets: A study on brand perception and decision-making. Acta Psychol..

[24] Nguyen N.M., Nguyen H.T., Cao T.A. (2024). Effects of social media marketing activities on perceived values, online brand engagement, and brand loyalty. Emerg. Sci. J..

[25] Baber R., Baber P. (2023). Influence of social media marketing efforts, e-reputation and destination image on intention to visit among tourists: Application of S-O-R model. J. Hosp. Tour. Insights.

[26] Shuyi J., Al Mamun A., Naznen F. (2024). Social media marketing activities on brand equity and purchase intention among chinese smartphone consumers during COVID-19. J. Sci. Technol. Policy Manag..

[27] Lusianingrum F.P.W., Pertiwi W.N.B. (2023). Applying Stimulus-Organism-Response (SOR) adoption for predicting generation Z’s intention to visit tourism in indonesia. Qubahan Acad. J..

[28] Rossmann C. (2002). Theory of Reasoned Action—Theory of Planned Behavior.

[29] Zhang X.-Y., Chao C.-T., Chiu Y.-T., Chen H.-S. (2024). Study of the Correlation between Streaming Video Platform Content on Food Production Processes and the Behavioral Intentions of Generation Z. Foods.

[30] Pham A.D., Dao T.T.T., Pham P.M., Pham Y.H., Nguyen H.T., Pham L.N. (2024). How Does Conformity Shape Influencer Marketing in the Food and Beverage Industry? A Case Study in Vietnam. J. Internet Commer..

[31] Daowd A., Hasan R., Eldabi T., Rafi-ul-Shan P.M., Cao D., Kasemsarn N. (2021). Factors affecting eWOM credibility, information adoption and purchase intention on Generation Y: A case from Thailand. J. Enterp. Inf. Manag..

[32] Konar R., Balasubramanian K., Kumar J. (2020). The Impact of Social Media on Consumers’ Purchasing Behaviour in Malaysian Restaurants. J. Spat. Organ. Dyn..

[33] Brodie R.J., Hollebeek L.D., Jurić B., Ilić A. (2011). Customer Engagement: Conceptual Domain, Fundamental Propositions, and Implications for Research. J. Serv. Res..

[34] Hasani V.V., Zeqiri J., Todorovik T., Jaziri D., Toska A. (2023). Digital Content Marketing and EWOM: A Mediational Serial Approach. Bus. Syst. Res. J..

[35] Bukhori I., Sukma N.P.Y., Bonita S., Yuniarty (2022). The Influence of Social Media Marketing on Customer Engagement and Its Impact on Customer’s Purchase Intention in CV. Event Hunter Indonesia. Proceedings of the 2022 7th International Conference on Business and Industrial Research (ICBIR).

[36] Hollebeek L.D., Glynn M.S., Brodie R.J. (2014). Consumer Brand Engagement in Social Media: Conceptualization, Scale Development and Validation. J. Interact. Mark..

[37] Naumann K., Bowden J., Gabbott M. (2020). Expanding customer engagement: The role of negative engagement, dual valences and contexts. Eur. J. Mark..

[38] Dessart L., Aldás-Manzano J., Veloutsou C. (2019). Unveiling heterogeneous engagement-based loyalty in brand communities. Eur. J. Mark..

[39] Kim J., Hwang J. (2022). Who is an evangelist? Food tourists’ positive and negative eWOM behavior. Int. J. Contemp. Hosp. Manag..

[40] Andrade C., Paguay E., Viteri J., Esparza F. (2023). El sistema de mercadeo en pymes de producción láctea, como factor de competitividad y propuesta de marketing digital. Código Científico Rev. Investig..

[41] Filieri R., Acikgoz F., Ndou V., Dwivedi Y. (2021). Is TripAdvisor still relevant? The influence of review credibility, review usefulness, and ease of use on consumers’ continuance intention. Int. J. Contemp. Hosp. Manag..

[42] Bushara M.A., Abdou A.H., Hassan T.H., Sobaih A.E.E., Albohnayh A.S.M., Alshammari W.G., Aldoreeb M., Elsaed A.A., Elsaied M.A. (2023). Power of Social Media Marketing: How perceived value mediates the impact on restaurant followers’ purchase intention, willingness to pay a premium price, and e-WoM?. Sustainability.

[43] Nyagadza B., Mazuruse G., Simango K., Chikazhe L., Tsokota T., Macheka L. (2023). Examining the influence of social media eWOM on consumers’ purchase intentions of commercialised indigenous fruits (IFs) products in FMCGs retailers. Sustain. Technol. Entrep..

[44] Park D.-H., Lee J., Han I. (2007). The Effect of On-Line Consumer Reviews on Consumer Purchasing Intention: The Moderating Role of Involvement. Int. J. Electron. Commer..

[45] Verma D., Dewani P.P., Behl A., Dwivedi Y.K. (2023). Understanding the impact of eWOM communication through the lens of information adoption model: A meta-analytic structural equation modeling perspective. Comput. Hum. Behav..

[46] Brodie R.J., Ilic A., Juric B., Hollebeek L. (2013). Consumer engagement in a virtual brand community: An exploratory analysis. J. Bus. Res..

[47] Chae H., Ko E., Han J. (2015). How do customers’ SNS participation activities impact on customer equity drivers and customer loyalty? Focus on the SNS services of a global SPA brand. J. Glob. Sch. Mark. Sci..

[48] Kristia K. (2021). Mediating Effect of Customer Engagement on the Relations between eWOM, Environmental Concern, and Intention to Purchase Second-hand Clothing among College Students in Yogyakarta. J. Manaj. Bisnis..

[49] Zahrah N., Ruzain M.F., Sengorou J.A., Mat Salleh N.S. (2024). The Impact of User-Generated Content and Electronic Word-of-Mouth on Consumer Purchase Intention: Consumer Engagement as a Mediator. Int. J. Acad. Res. Bus. Soc. Sci..

[50] Garzón G., Ruiz G., Juárez B. (2020). Análisis de la confianza, lealtad e intención de compra digital de los consumidores post-millennials. Rev. Espac..

[51] Lima Vargas Á.E., Cervantes Aldana F.J., Lima Vargas S. (2021). La comunicación en redes sociales por parte de las organizaciones como influencia en el comportamiento co-creador de valor en el consumidor generación Z. Diálogos Para la Investigación en Comunicación, Educación y Tecnologías.

[52] Lima Vargas S., Lima Vargas Á.E. (2023). La participación en redes sociales de las empresas del mercado de la moda desde la perspectiva de la generación Z. Contaduría Y Adm..

[53] de Sousa Pereira M.J., Cardoso A., Canavarro A., Figueiredo J., Garcia J.E. (2023). Digital Influencers’ Attributes and Perceived Characterizations and Their Impact on Purchase Intentions. Sustainability.

[54] Li Z., Chan C., Chen Y.-F., Chan W.W.H., Im U.L. (2024). Millennials’ Hotel Restaurant Visit Intention: An Analysis of Key Online Opinion Leaders’ Digital Marketing Content. J. Qual. Assur. Hosp. Tour..

[55] Sanmiguel P., Sadaba T. (2024). Who is Accountable for the Negative Effects of Influencer Marketing? Voices of the Influencer Ecosystem. Rev. ICONO 14 Rev. Científica Comun. Tecnol. Emerg..

[56] Núñez-Gómez P., Sánchez-Herrera J., Pintado-Blanco T. (2020). Children’s Engagement with Brands: From Social Media Consumption to Brand Preference and Loyalty. Sustainability.

[57] Serman Z., Sims J. (2020). How social media influencers affect consumers purchase habit?. Proceedings of the UK Academy for Information Systems Conference 2020, Oxford, UK, 29 April 2020.

[58] Magano J., Au-Yong-Oliveira M., Walter C.E., Leite Â. (2022). Attitudes toward Fashion Influencers as a Mediator of Purchase Intention. Information.

[59] Folkvord F., Roes E., Bevelander K. (2020). Promoting healthy foods in the new digital era on Instagram: An experimental study on the effect of a popular real versus fictitious fit influencer on brand attitude and purchase intentions. BMC Public Health.

[60] Popy N.N., Bappy T.A. (2022). Attitude toward social media reviews and restaurant visit intention: A Bangladeshi perspective. S. Asian J. Bus. Stud..

[61] Arratia Mendoza M.L., Sánchez Tovar Y., Mendoza Flores J.E. (2024). The influence of social networks recommendations on the purchase intention: A comparative study between the millennial and centennial generation. Paakat Rev. Tecnol. Soc..

[62] Anber Mohammad A.M. (2022). The Impact of Digital Marketing Success on Customer Loyalty. Mark. Manag. Innov..

[63] Li C.H., Chan O.L.K., Chow Y.T., Zhang X., Tong P.S., Li S.P., Ng H.Y., Keung K.L. (2022). Evaluating the Effectiveness of Digital Content Marketing Under Mixed Reality Training Platform on the Online Purchase Intention. Front. Psychol..

[64] Wangpo K., Wangmo S. (2022). The influence of social media marketing on purchase intention: The mediating role of brand equity. Asian J. Res. Mark..

[65] Spence C., Okajima K., Cheok A.D., Petit O., Michel C. (2016). Eating with our eyes: From visual hunger to digital satiation. Brain Cogn..

[66] Yadav M., Rahman Z. (2017). Measuring consumer perception of social media marketing activities in e-commerce industry: Scale development & validation. Telemat. Inform..

[67] Liu X., Shin H., Burns A.C. (2021). Examining the impact of luxury brand’s social media marketing on customer engagement: Using big data analytics and natural language processing. J. Bus. Res..

[68] Akar E., Topçu B. (2011). An Examination of the Factors Influencing Consumers’ Attitudes Toward Social Media Marketing. J. Internet Commer..

[69] Zhang L., Akbar S., Tomuș A.M., Solomon A.G. (2022). Antecedents and Consequences of Banking Customers’ Behavior towards Social Media: Evidence from an Emerging Economy. Behav. Sci..

[70] Sijabat D.C.S., Saputra F.D., Ikhsan R.B., Yuniarty (2020). The Impact of Social Network Marketing and Customer Engagement on Purchase Intentions in Wedding Service Business. Proceedings of 2020 International Conference on Information Management and Technology (ICIMTech), Bandung, Indonesia, 13–14 August 2020.

[71] Jang J.A., Kim J.-M., Jung H. (2024). Impact of Social Media Use on Segmentation of Dining out Behavior Among Younger Generations: A Case Study in South Korea. Foods.

[72] Abergos L.A.G., Chua I.E., Dela Paz M.J., Galvin L.C.D., Gatbonton G.A., Grimaldo H.C.O., Mayo S.A.V., Tumbali M.V.L. (2024). The effectiveness of social media as a marketing tool for selected MSMES coffee shops in Manila. Glob. Sci. Acad. Res. J. Econ. Bus. Manag..

[73] Băltescu C.A., Untaru E.-N. (2024). Exploring the Characteristics and Extent of Travel Influencers’ Impact on Generation Z Tourist Decisions. Sustainability.

[74] Tecnológico de Monterrey (2024). La Generación Z Y Sus Hábitos de Compra.

[75] Cruz-Estrada I., Miranda-Zavala A.M. (2020). Redes Sociales Digitales en la comunicación con los consumidores de restaurantes de la zona gastronómica de Tijuana. Estud. Soc. Rev. Aliment. Contemp. Desarro. Reg..

[76] Creswell J.W. (2022). A Concise Introduction to Mixed Methods Research.

[77] Chetioui Y., Benlafqih H., Lebdaoui H. (2020). How fashion influencers contribute to consumers’ purchase intention. J. Fash. Mark. Manag..

[78] Durukan T., Bozaci I. (2012). A Survey on determinants of Word of Mouth in Social Media. Int. J. Econ. Manag. Sci..

[79] Hernández Sampieri R., Collado C.F., del Pilar Baptista Lucio D.M. (2014). Metodología de la Investigación.

[80] Brown T.A. (2015). Confirmatory Factor Analysis for Applied Research.

[81] Hooper D., Coughlan J., Mullen M.R. (2008). Structural Equation Modelling: Guidelines for Determining Model Fit. Electron. J. Bus. Res. Methods.

[82] Hair J.F., Hult G.T.M., Ringle C.M., Sarstedt M. (2017). A Primer on Partial Least Squares Structural Equation Modeling (PLS-SEM).

[83] Cohen J. (1988). Statistical Power Analysis for the Behavioral Sciences.

[84] Hernández Lalinde J.D., Espinosa Castro F., Rodríguez J.E., Chacón Rangel J.G., Toloza Sierra C.A., Arenas Torrado M.K., Carrillo Sierra S.M., Bermúdez Pirela V.J. (2018). Sobre el uso adecuado del coeficiente de correlación de Pearson: Definición, propiedades y suposiciones. Arch. Venez. Farmacol. Ter..

[85] Fornell C., Larcker D.F. (1981). Evaluating Structural Equation Models with Unobservable Variables and Measurement Error. J. Mark. Res..

[86] Azman A.B., Majid M.A.A., Zainozaman M.S., Zulkifly M.I., Mahusain M.A. (2025). Scroll, Click, Buy: The Impact of Social Media Attributes on Purchase Intentions among Young Adults. J. Inf. Technol. Manag..

[87] Sun Y., Ly T.P. (2023). The Influence of Word-of-web on Customers’ Purchasing Process: The Case of Xiaohongshu. J. China Tour. Res..

[88] Flores-Rueda I.C., Espinosa-Delgado J.M., Torres-Rivera M.P. (2021). Aplicaciones de Herramientas de Mercadotecnia: Análisis de Oportunidades de Mercado, Formación de Negocios, Estrategias Digitales y Comercialización.

[89] Chen X., Chen Z. (2025). Impact of video content on gastronomic image construction and tourists’ intention to (re-) visit Macao. Tour. Recreat. Res..

[90] Horton D., Wohl R.R. (1956). Mass Communication and Para-Social Interaction: Observations on Intimacy at a Distance. Psychiatry.

[91] Lacap J.P.G., Cruz M.R.M., Bayson A.J., Molano R., Garcia J.G. (2024). Parasocial relationships and social media interactions: Building brand credibility and loyalty. Span. J. Mark. ESIC.

[92] Bhattacharya A. (2023). Parasocial Interaction in Social Media Influencer-Based Marketing: An SEM Approach. J. Internet Commer..

[93] Labrecque L.I. (2014). Fostering Consumer–Brand Relationships in Social Media Environments: The Role of Parasocial Interaction. J. Interact. Mark..

[94] Vázquez-Medina J.A., Medina F.X. (2020). Traditional Mexican Cuisine: Heritage Implications for Food Tourism Promotion. Gastron. Tour..

[95] Morales-Guerrero J.C., Miranda-Alatriste P.V., Villafuerte-Salazar M.G., Espinosa-Cuevas Á., Cassis-Nosthas L., Colín-Ramírez E. (2023). Determination of the chemical compositions of Mexican antojitos and dishes in Mexico City. J. Food Compos. Anal..

[96] Unar-Munguía M., Cervantes-Armenta M.A., Rodríguez-Ramírez S., Bonvecchio Arenas A., Fernández Gaxiola A.C., Rivera J.A. (2024). Mexican national dietary guidelines promote less costly and environmentally sustainable diets. Nat. Food.

[97] Curi-Quinto K., Unar-Munguía M., Rodríguez-Ramírez S., Rivera J.A., Fanzo J., Willett W., Röös E. (2022). Sustainability of Diets in Mexico: Diet Quality, Environmental Footprint, Diet Cost, and Sociodemographic Factors. Front. Nutr..

[98] Ibarrola-Rivas M.J., Galicia L. (2017). Repensando la seguridad alimentaria en México: La necesidad de discutir políticas transversales sustentables enlazando la producción de alimento con el consumo. Investig. Geogr..

[99] Pineda E., Barbosa Cunha D., Taghavi Azar Sharabiani M., Millett C. (2023). Association of the retail food environment, BMI, dietary patterns, and socioeconomic position in urban areas of Mexico. PLoS Glob. Public Health.

